# Portomesenteric Reconstruction during Whipple Procedure Using Autologous Left Renal Vein Patch Graft in a Patient with a Gastric Cancer Recurrence

**DOI:** 10.1155/2023/2717041

**Published:** 2023-04-27

**Authors:** Lidija Ljubicic, Igor Petrovic, Andrea Crkvenac Gregorek, Hrvoje Silovski

**Affiliations:** ^1^Department for Respiratory Diseases Jordanovac, University Hospital Centre Zagreb, Zagreb, Croatia; ^2^Department of Surgery, University Hospital Centre Zagreb, Zagreb, Croatia; ^3^School of Medicine, University of Zagreb, Zagreb, Croatia

## Abstract

The case of vascular reconstruction of the superior mesenteric and portal vein confluence using a left renal vein (LRV) graft has been researched in this paper. The patient was a 66-year-old female who presented with features of biliary obstruction. A contrast-enhanced computed tomography scan revealed bile duct dilatation and a common bile duct tumor mass. Four years ago, she underwent stomach resection with subsequent Billroth II gastrojejunostomy due to gastric cancer. After surgical resection, on histopathological and immunohistochemistry examination, a recurrence of previously resected poorly cohesive gastric cancer was found.

## 1. Introduction

The pancreas is an unusual site for solitary metastasis, compared to other primary cancers. Therefore, data regarding the surgical outcome of pancreatic resections performed for metastases from different primary tumors are limited. Nevertheless, pancreaticoduodenectomy (PD) for metastatic disease to the pancreas should be considered a treatment option in appropriately selected patients with isolated metastases to the pancreas. In the past, major vascular involvement was considered a contraindication of PD. Nowadays, we witness the continuous advancement of surgical techniques and procedures, alongside the progress of surgeons' experience, so it comes to no surprise that PD with venous reconstruction has become the standard of care in the treatment of pancreatic cancer. The same applies to metastatic lesions in the pancreas, so pancreatic metastasectomy should always be considered among other therapeutical options. Precisely with this, the term borderline resectable tumor was developed and defined by the National Comprehensive Cancer Network (NCCN) in 2006. According to NCCN, the term “borderline resectable” implies a group of patients at a high risk for margin positive resection. In those patients, administration of neoadjuvant therapy should be considered [1]. The most recent NCCN guidelines outline a definition of “borderline resectable” as a tumor demonstrating radiographic contact, with superior mesenteric vein–portal vein (SMV–PV) of >180 or contact ≤180 with contour irregularity of the vein or thrombosis of the vein but with suitable vessel proximal and distal to the site of involvement [1]. However, studies have shown that the survival of patients whose tumors involve SMV/PV and who undergo PD with vascular resection does not differ from those who undergo standard PD [2]. A recent meta-analysis of 32 studies evaluating mortality, morbidity, and long-term survival of pancreatic resections, both with and without venous resection, has revealed comparable rates of complications, reoperations, and overall survival in patients with and without vascular resection [3]. In contrast to venous resection, few studies have investigated the outcome of pancreatectomy associated with artery resection and there is still no clear consensus. Del Chiaro et al. provided a retrospective analysis of a cohort of operated borderline or locally advanced pancreatic cancer patients, with surgically confirmed arterial involvement. Both short and long-term outcomes were analyzed and compared in patients who underwent pancreatectomy associated with artery resection and patients who underwent palliative surgery. This study showed that there were no differences in postoperative mortality (2.9% vs. 2.6%, *p* = 0.9) and postoperative surgical complications with an advantage of survival compared to palliation [4].

However, in cases with evident arterial invasion, patients should primarily be treated by neoadjuvant therapy and then reevaluated for possible surgery afterward [5].

Depending on the degree of vascular involvement, different techniques for resection and reconstruction can be used. International Study Group of Pancreatic Surgery divided the venous resection into four types, determined by the performed reconstruction: venorrhaphy, patch, primary anastomosis, and interposition conduit [6]. Both synthetic and autologous grafts have been used. The use of the left renal vein (LRV) for autologous grafting offers some advantages; it provides a graft with a suitable length, well matched diameter, and it is easily accessible. According to several studies, it causes no significant impairment on renal function [7]. Some other veins could be selected as autologous vein grafts for vascular reconstruction, such as a saphenous, iliac, gonadal, femoral, jugular, and umbilical vein [7–9].

The aim of this report is to describe the use of the LRV as a conduit for venous reconstruction, after the Whipple procedure in a patient with gastric cancer recurrence involving the head of the pancreas, thus causing common bile duct obstruction.

## 2. Case Report

A 66-year-old female was admitted to our hospital due to jaundice. She has experienced right upper quadrant abdominal pain, paired with nausea and bloating for several weeks prior. Moreover, the patient also reported a 13-pound weight loss over the past 4 months. The patient had been diagnosed with gastric cancer 4 years ago. Stomach resection was done with subsequent Billroth II gastrojejunostomy. The histopathological examination of the specimen revealed a poorly cohesive adenocarcinoma (diffuse type according to Lauren) that extended to the subserosa and omentum, then spreading to the bulb of the duodenum, infiltrating all layers of the wall. Resection margins were not involved (R0), and in 12 isolated lymph nodes, no signs of metastasis (N0) leading to pathological staging of pT4aN0 were found. Additional immunohistochemical analysis showed that the tumor cells were positive for AE1/AE3 (human anion exchangers 1 and 2) and negative for synaptophysin. Ki-67 (proliferation marker) was 32%. The patient underwent chemoradiotherapy postoperation. Physical examination showed signs of skin and scleral jaundice. Initial laboratory tests indicated the following: hemoglobin, 135 g/L (norm: 119–157 g/L); albumin, 39.7 g/L (norm: 40–55 g/L); total bilirubin, 55 *μ*mol/L (norm: 3–20 *μ*mol/L); direct bilirubin, 44 *μ*mol/L (norm: <5  *μ*mol/L); alkaline phosphatase, 1134 U/L (norm: 60–142 U/L); gamma-glutamyl transferase, 1502 U/L (norm: 11–55 U/L); and alanine aminotransferase, 493 U/L (norm: 12–48 U/L). A tumor marker CA19-9 was elevated, measuring 181.1  U/mL (norm: <37 kIU/L ). Contrast-enhanced abdominal computed tomography was requested for further evaluation and showed significantly dilated intra- and extrahepatic bile ducts in both liver lobes. In addition, the distal segment of the common bile duct was narrowed in a length of 2 cm due to increased vascularization of the wall, which primarily indicated the bile duct's tumor process. The liver had no evident focal lesions, and there were no signs of tumor in the area of previously performed gastrojejunal anastomosis. Upper gastrointestinal endoscopy did not detect lesions; the biopsy specimen contained no tumor tissue.

**Figure 1:**
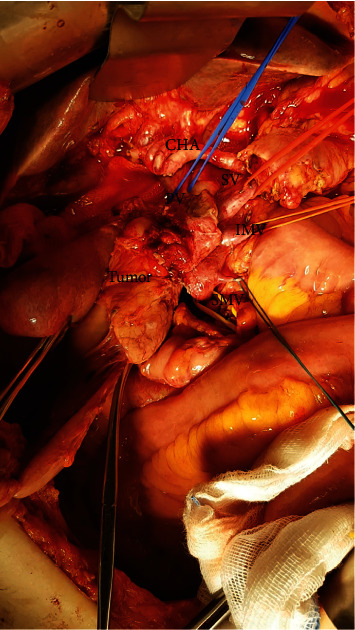
Dissection around the common hepatic artery (CHA). The portal vein (PV), splenic vein (SV), inferior mesenteric vein (IMV), and superior mesenteric vein (SMV) are taped individually. The tumor was left adherent on the PV–SMV axis afterward the artery first approach.

The patient underwent a cephalic PD (Whipple procedure). A complete medial laparotomy was done. After adhesiolysis and omentectomy, anterograde cholecystectomy was performed with the hepatic duct encircled near the cystic duct junction. Resection of the common hepatic duct, gastroduodenal artery, pancreatic neck, the first loop of jejunum, and resection of the mesopancreas using artery first approach was performed. Finally, the specimen was attached only on the right circumference of the PV–SMV axis (Figure 1). Due to partial tumor infiltration of the vein, the decision was made to perform partial vein resection and reconstruction using an autologous LRV.

**Figure 2:**
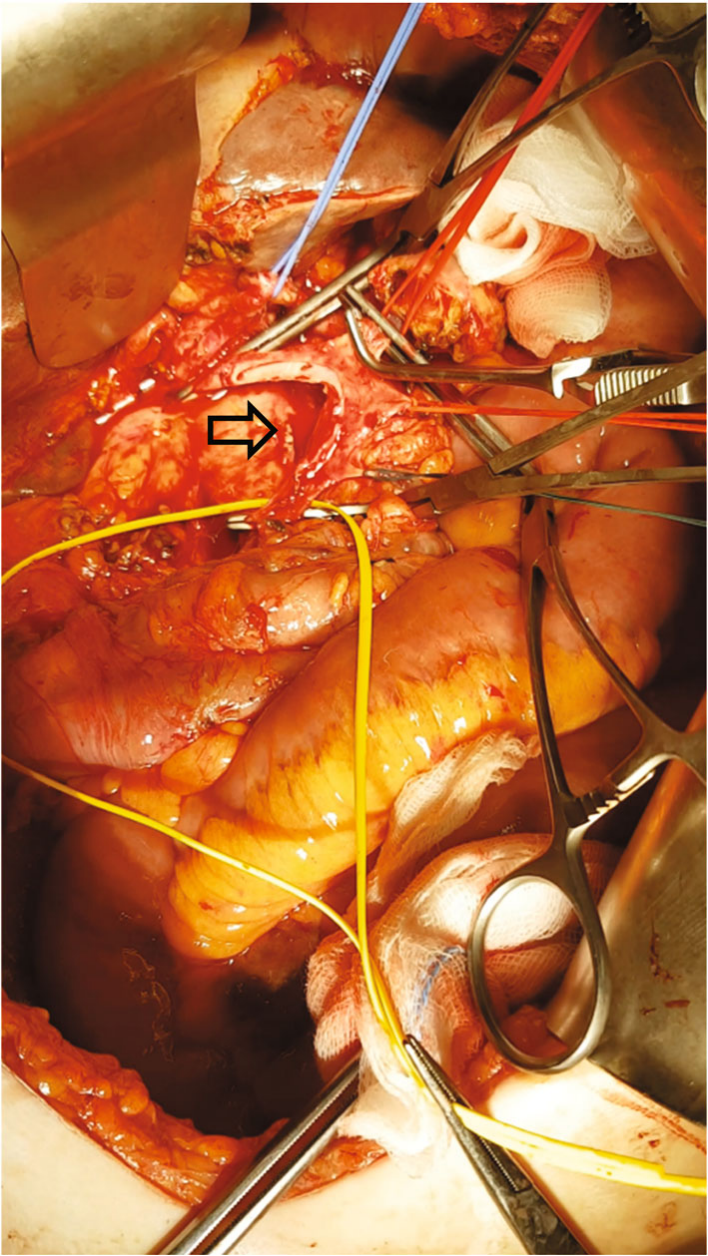
Arrow indicates the resected segment of the PV–SMV axis.

Vascular clamps were used to control the superior mesenteric vein, inferior mesenteric vein, splenic vein, and portal vein before resection of the involved venous segment (Figure 2).

**Figure 3:**
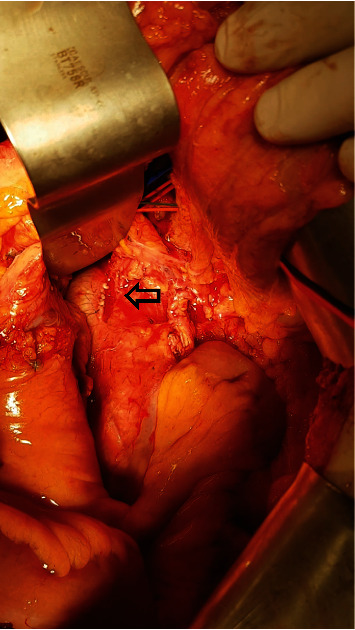
Operative finding. Sutured inferior vena cava (IVC) after left renal vein (LRV) harvesting (arrow).

**Figure 4:**
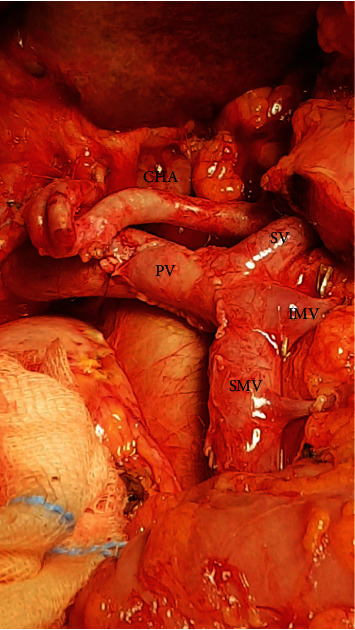
Intraoperative finding. A segment of the PV–SMV axis was removed and reconstructed using an LRV patch. CHA: common hepatic artery; SV: splenic vein; IMV: inferior mesenteric vein; SMV: superior mesenteric vein; PV: portal vein.

Circumferential LRV resection was carried out after subsequent venous clamping, proximally at the confluence with the inferior vena cava, and distally, before the junction of the adrenal, lumbar, and left ovarian veins, to enable venous drainage of the left kidney via collateral venous blood flow (Figure 3). In doing so, we obtained 4 cm LRV graft. The LRV was opened longitudinally and used as a “patch” graft to reconstruct the partially resected PV–SMV axis (Figure 4). The inflow of the left splenic vein was left intact. This was performed using polypropylene 6/0 suture. Gastrointestinal reconstruction was performed in a standard way, using Blumgart pancreaticojejunal anastomosis after the completion of venous reconstruction.

Specimen's gross examination revealed infiltration of the distal part of the common bile duct, the anterior wall of the pancreatic head, the superior mesenteric vein, and the portal vein in a length of 4 cm in the extent of one-third of the right circumference of the vein. The tumor measured 50 × 40 mm.

Upon histological analysis, whitish solid tumor tissue was noted, which infiltrated the proximal part of the gallbladder, the bile duct, the surrounding blood vessels, part of the wall of the duodenum up to the level of the Vateri papilla, and the largest part of the head of the pancreas. By examining a thin slice (section), tumor tissue composed of aggregates, streaks, and individual atypical epithelial cells, partly of the signet ring type, was observed.

Five out of thirteen lymph nodes were positive for tumor infiltration: two in the peripancreatic adipose tissue, two in the hepatoduodenal ligament, and one along the cystic duct.

The further postoperative course was uneventful, and the patient was discharged in stable condition on the 13th postoperative day. The postoperative serum creatinine value was 31 *μ*mol/L (normal range 49–90 *μ*mol/L). CT scan 6 months postoperatively showed no evidence of recurrent malignancy with patency of the venous graft. Unfortunately, the patient died during follow-up from recurrent disease 17 months after the second operation and 65 months after the primary operation.

## 3. Discussion

Pancreatic ductal adenocarcinoma (PDAC) and primary bile duct cancer (cholangiocarcinoma) are the two most common causes of malignant biliary obstruction [10]. Other causes include ampullary carcinoma, primary duodenal adenocarcinoma, and pancreatic neuroendocrine tumors. Malignant biliary obstruction associated with gastric cancer is not common, with incidence reported to be from 1.3% to 2.3% [11].

Surgical resection represents the only potential therapeutic modality for early and some advanced forms of gastric cancer. For many years, there have been controversies regarding the optimal surgical treatment of gastric cancer. However, using evidence-based principles, gastrectomy with extensive lymph node dissection has been reported to improve long-term survival.

On the contrary, the clinical outcomes of PD for locally advanced gastric cancer remain unclear.

In 2019, Li et al. provided a systematic review and pooled analysis of relevant data in the literature regarding the clinical outcome of PD for locally advanced gastric cancer invading the duodenum and/or pancreas. A total of 13 articles involving 69 patients were analyzed. Overall 5-year survival and median survival were 39.3% and 26 months, respectively, with positive peritoneal lavage cytology as the only independent prognostic factor for the poor outcome at multivariate analysis [12].

In the retrospective study done by Wang et al., 17 patients (32%) underwent total gastrectomy (TG) or distal subtotal gastrectomy (SG) combined with PD simultaneously. The actual 1- and 3-year survival rates after resection were 77% and 34%, respectively, and three patients survived for more than 5 years after surgery. In addition, the tumor-free resection margin (*P* = 0.0174) and a well-differentiated histologic type (*P* = 0.0011) were significant prognostic factors in univariate analysis [13].

Lee et al. analyzed 25 patients who underwent PD with gastrectomy due to suspicion of direct PD segment involvement or enlargement of lymph nodes around the pancreas head. The median survival was 16.5 months, with a 5-year survival rate of 15.8%. Two patients with T2bN0M0 and T2bN1M0 stages were alive for 11.5 years and 5.7 years without any evidence of cancer recurrence. Postoperative complications were encountered in eight patients (32%), but re-operation was required only in two cases [14].

In addition to gastric cancer, malignant biliary obstruction can also develop as a result of metastasis from distant cancers, such as colorectal cancer, renal cancer, lung cancer, breast cancer, lymphoma, and malignant melanoma [15]. Renal cell carcinoma appears to be the most common primary tumor to cause secondary pancreatic tumors [16]. In large autopsy surveys provided by Z'graggen et al., the prevalence of metastasis to the pancreas was as high as 11% [17]. Sperti et al. performed a review of the published literature, concentrating on the early and long-term results of surgery for the most frequent primary tumors metastasizing to the pancreas. The advantage of metastasectomy in terms of patient survival has been observed for metastases from renal cell cancer, while for other primary tumors, such as lung and breast cancers, the role of surgery is mainly palliative [18].

The benefit of resection for pancreatic metastases is largely dependent on the tumor biology of the primary cancer; renal cell cancer is associated with the best outcome of a 5-year survival rate, greater than 70% [19]. These conditions are sometimes difficult to distinguish from primary pancreatobiliary tumors, based only on radiological findings. The optimal treatment regime remains controversial, without a clear consensus. Regardless of the cause, metastasectomy with R0 resection should be attempted whenever possible, as it only offers the best chance for a cure. Poletto et al. recently published the first case of curative surgery with the radical intent of recurrent gastric cancer causing a malignant biliary obstruction. Pancreatoduodenectomy with regional lymphadenectomy was performed [20].

To achieve R0 margins, simultaneous resection of the SMV–PV confluence may be performed safely during PD. Although current preoperative imaging techniques can detect vascular tumor involvement, this is often an intraoperative finding, with decision-making regarding venous resection and reconstruction.

Several types of venous resection and reconstruction have been described according to the type and length of the venous resection. Type 1: partial venous excision with direct closure (venorrhaphy) by suture closure; type 2: partial venous excision using a patch; type 3: segmental resection with primary venovenous anastomosis; and type 4: segmental resection with interposed venous conduit and at least two anastomoses [21]. The length of the vein segment affected by the tumor is critical for vein resection and reconstruction. However, there is no clear consensus on which size of the defect end-to-end anastomosis can be performed safely without margin tension. According to some studies, reconstruction using a graft is required in cases in which a venous resection is ≥ 31 mm [22]. In contrast, other reports show that even in cases with a gap of 50 mm and more end-to-end anastomosis is possible [23–25]. Maneuvers like full hepatic release and mobilization with right and left portal trunk dissection, and complete mobilization of the mesenteric root distally, are performed to secure more centimeters of length [18].

There are several vascular reconstruction techniques, and the optimal method is the one that provides the most significant opportunity for a safe R0/R1 resection of the tumor. Various types of grafts have been described for venous reconstruction; however, the decision for the optimal type depends on several factors, such as the vessel lumen, the type of vascular reconstruction (patch plastic or a tube substitute), the septic contamination of the operative field, the accessibility of the graft, and the surgeon's experience [26].

The decision on the most favorable method predominantly depends on the intraoperative finding. In a multicenter study provided by Ravikumar et al., a total of 229 patients underwent portal vein resection; 129 (56·3%) underwent primary closure, 64 (27·9%) had an end-to-end anastomosis, and 36 (15·7%) had an interposition graft. The surgical morbidity and mortality in that study were comparable in all three cohorts [27].

In our case, vein involvement by the tumor was not detected preoperatively, but during the resection. As the SMV–PV confluence defect was 4 × 1 cm, the LRV graft seemed ideal for reconstruction for several reasons; the vein was the same length as a SMV–PV defect, in the same operative field, autologous, and of similar caliber as the portal vein. The use of autologous grafts has particular advantages, such as lower risk of infection or thrombosis, compared with the use of synthetic materials [28]. The data from the review done by Labori et al. showed that graft thrombosis was more likely after synthetic graft reconstruction, but an association with increased mortality was not shown [29].

In a study done by Roch et al., pancreatectomy with portal vein resection was performed in 220 patients, of which 36 (16.4%) developed thrombosis after a median of 15.5 days. The rate of SMV/PV thrombosis varied according to SMV/PV reconstruction technique: 12.8% after venorrhaphy, 13.2% end-to-end anastomosis, 22.6% autologous vein, and 83.3% synthetic graft interposition [30]. Recommendations for anticoagulation following major venous reconstruction for pancreatic adenocarcinoma (PA) are not clearly established. In a meta-analysis provided by Chandrasegaram et al. that evaluated the effectiveness of postoperative anticoagulation, early PV thrombosis was similar in patients who did and did not receive anticoagulation [31].

There are some controversies regarding renal function deterioration after obtaining LRV graft without its reconstruction. The risk of such an outcome is minimized since LRV has several collateral branches (gonadal and azygos vein) that drain venous return from the left kidney [32].

Other autologous vein graft options include the internal jugular vein, femoral vein, saphenous, iliac, splenic, and also gonadal vein [8, 9, 33]. A splenic vein for a PV/SMV reconstruction can be used as an autologous interposition graft or in the turndown technique, which is used in high-risk patients for an anastomotic leak (long-term corticosteroids or immunosuppressant therapy). Clout et al. described the first report in the literature on the splenic vein turndown technique for SMV reconstruction following PD and venous resection for pancreatic malignancy. This technique, mainly used in trauma repair, preserves the splenic–portal vein confluence and utilizes the proximal splenic vein to anastomose the jejunal and ileal branches [34]. One of the limitations of this technique is the concomitant splenectomy needed to prevent segmental portal hypertension and gastric varices. Matsui and Takigawa published the use of autologous splenic veins without reconstruction of splenic vein-portal vein (SPV–PV) confluence. They proved the absence of left-sided portal hypertension due to preservation of the left gastroepiploic, left gastric, and posterior gastric veins. They also proved pathohistologically tumor negative margin of SPV graft before using it for PV/SMV reconstruction [35].

The graft can also be sourced from a cadaveric donor vessel. Jugular vein is widely used as an autologous graft because of its good size match to the portal vein. Since vascular resection and reconstruction were unplanned, the use of the jugular vein in our case would require an additional incision. Another operation field is also needed when using lower extremity veins (femoral or saphenous) with the risk of postoperative venous insufficiency, surgical site infection, and also deep vein thrombosis [36].

Regarding “patch” reconstruction, in the last decade, a PPP (parietal peritoneum patch) was used if the vessel wall defect was less than 30% of its circumference; otherwise, a tube graft was preferred. PPPs were harvested from an area of the abdominal wall with intact peritoneum before clamping the vein (left or right hypochondrium), and the peritoneal surface is turned to the vessel lumen. Compared to autologous venous grafts, this technique has several advantages. The PPPs graft is readily available, operation time is not significantly prolonged, and there is no need for additional surgical procedures as in the case of harvesting another autologous vessel. In addition, there is no graft size limitation [26].

The safety of using the LRV as an autologous graft, in particular, concerning renal function, has been confirmed in several studies. For example, Smoot et al. described outcomes for nine patients undergoing PD with venous reconstruction. In eight patients, the LRV was used as an interposition graft, and in one patient, as a patch graft. Through the follow-up period (mean was 6.8 months), normal creatinine values were observed [8]. In a study provided by Suzuki et al., 14 patients underwent vascular reconstruction using a LRV graft, without adverse effects on early and long-term renal function [37]. In our case, creatinine levels remained normal both in the early and late postoperative course. If the adrenal and gonadal veins are preserved, no significant renal dysfunction is to be expected.

To the best of our knowledge, this is the first case of PD with venous resection and reconstruction using LRV in radical treatment of gastric cancer recurrence. Malignant biliary obstruction due to metastases from other primary sites is an uncommon condition. It is important to suspect them in cases with a positive history of malignant diseases.

## 4. Conclusion

Vascular resections are performed to obtain R0 margins during pancreatoduodenectomy. High-quality axial imaging with three-dimensional vascular reconstructions helps to assess the tumor–vessels relationship in preoperative planning. Vascular involvement by tumors is often discovered during the operation, and in such cases, reconstruction using the LRV graft offers significant advantages over other reconstruction options. A multidisciplinary team approach in preoperative planning and during the operation plays a prominent role in cancer treatment.

## Data Availability

The data is available in hospital's IT system.
